# SERS Amplification in Au/Si Asymmetric Dimer Array Coupled to Efficient Adsorption of Thiophenol Molecules

**DOI:** 10.3390/nano11061521

**Published:** 2021-06-08

**Authors:** Grégory Barbillon, Andrey Ivanov, Andrey K. Sarychev

**Affiliations:** 1 EPF-Ecole d’Ingénieurs, 3 bis Rue Lakanal, 92330 Sceaux, France; 2 Institute for Theoretical and Applied Electrodynamics, Russian Academy of Sciences, 125412 Moscow, Russia; av.ivanov@physics.msu.ru (A.I.); sarychev@bioplasmonics.com (A.K.S.)

**Keywords:** SERS, sensors, dimer, gold, silicon, adsorption

## Abstract

Maximizing the surface-enhanced Raman scattering (SERS) is a significant effort focused on the substrate design. In this paper, we are reporting on an important enhancement in the SERS signal that has been reached with a hybrid asymmetric dimer array on gold film coupled to the efficient adsorption of thiophenol molecules on this array. Indeed, the key factor for the SERS effect is the adsorption efficiency of chemical molecules on the surface of plasmonic nanostructures, which is measured by the value of the adsorption constant usually named *K*. In addition, this approach can be applied to several SERS substrates allowing a prescriptive estimate of their relative performance as sensor and to probe the affinity of substrates for a target analyte. Moreover, this prescriptive estimate leads to higher predictability of SERS activity of molecules, which is also a key point for the development of sensors for a broad spectrum of analytes. We experimentally investigated the sensitivity of the Au/Si asymmetric dimer array on the gold film for SERS sensing of thiophenol molecules, which are well-known for their excellent adsorption on noble metals and serving as a proof-of-concept in our study. For this sensing, a detection limit of 10 pM was achieved as well as an adsorption constant *K* of 6 × 10${}^{6}$ M${}^{-1}$. The enhancement factor of 5.2 × 10${}^{10}$ was found at the detection limit of 10 pM for thiophenol molecules.

## 1. Introduction

During the past decade, the design of Surface-Enhanced Raman Scattering (SERS) substrates with high-enhancement factors (EF) is an important point for biological and chemical sensing [1,2,3,4]. The plasmonic nanostructures allow confining the electric field in the nanoscale zones called hotspots in order to maximize the EF factor [5]. These hotspots can be obtained by adjusting the geometry of plasmonic nanostructures as well as the nature of plasmonic materials [5]. This adjustment can be achieved by lithographic techniques, such as electron beam lithography [6,7,8], nanoimprint lithography [9,10,11], nanosphere lithography [12,13], and optical lithography [14,15,16,17]. Several designs have already been explored, such as nanorods, nanodisks, and nanodimers, allowing high EF values (EF = 10${}^{6}$–10${}^{9}$) [18,19]. Among these latter, the nanodimers as disk dimers separated by a nanogap enable a high field enhancement within the nanogap and thus to obtain a sensitive detection of various molecules up to the single molecule level [20]. Another way for enhancing the Raman signal of molecules is to realize the plasmonic nanostructures on a metallic film, which increases the enhancement factor by 1 or 2 magnitude orders due to a coupling between the nanosystems via surface plasmon polaritons or hybridization of localized plasmonic modes with the image modes in a plasmonic film [21,22,23,24]. An alternative direction for improving the Raman signal is to use hybrid nanostructures based on metal (gold, silver) and semiconductor (silicon, zinc oxide). In these types of nanostructures, hotspots can emerge at the interface between the semiconductor and the metal, and thus enhancing the Raman signal of molecules achieving EF values in the following range 10${}^{6}$–10${}^{10}$[25,26,27,28,29,30,31,32]. Moreover, several groups have already studied Au/Si nanostructures for thiophenol detection by SERS [33,34,35,36,37]. However, these works did not take into account the adsorption constant in their calculation of the enhancement factor. This adsorption constant is another aspect to be taken into account for obtaining a SERS spectrum, and corresponds to the efficiency of adsorption of the molecules on the surface of the plasmonic nanostructures. This adsorption constant (named *K*) is determined from the Langmuir model [38,39,40,41].

The aim of this paper is to present a very sensitive detection of chemical molecules (here thiophenol) with a design based on Au/Si asymmetric dimer array deposited on gold film. The design will enable obtaining: (i) strong electric fields within the nanogap of a dimer composed of two disks with different diameters; (ii) a reduction of the number of chemical molecules detected by using the structure of an asymmetric dimer and by the fact that the molecules of interest (here thiophenol) are only grafted on the gold part of the hybrid asymmetric dimers; (iii) an efficient adsorption of thiophenol molecules on the metallic surface of these hybrid plasmonic nanostructures.

## 2. Experimental Details

### 2.1. Fabrication and Simulations of the Asymmetric Dimers

**Figure 1:**
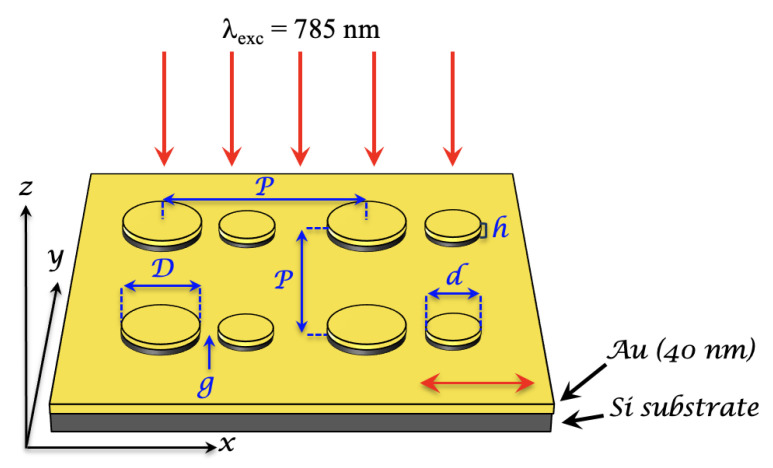
Design scheme of the Au/Si asymmetric dimer array on gold film (thickness = 40 nm) with all the paramaters of nanodimers: *D* = 130 nm, *d* = 65 nm, *P* = 300 nm, *g* = 10 nm and *h* = 40 nm (Si+Au). The red arrows correspond to the illumination by a laser at 785 nm under normal incidence, and at the bottom right, the double red arrow corresponds to the polarization direction of the laser oriented along the dimer axis.

The Au/Si asymmetric dimer array fabrication is composed by the following steps. The first step is the deposition of a gold layer (40 nm) by electron-beam evaporation (EBE) under normal incidence on a Si substrate on which is already deposited a 2-nm layer of Ti serving adhesion layer for Au. The second step consists of a deposition of a 90 nm layer of PMMA (polymethylmethacrylate A2) by spin-coating on gold film. Then, ten Au/Si asymmetric dimer arrays (300 × 300 µm^2^) are obtained by electron beam lithography followed by a development of the sample in a solution of 1:3 methylisobutylketone/isopropanol (MIBK/IPA) in order to reveal asymmetric dimer arrays. Next, two successive depositions of a 20 nm silicon layer and a 20 nm gold layer are gotten by EBE. The deposition speeds are set at 0.05 nm/s, 0.1 nm/s and 0.3 nm/s for Ti, Si and Au layers, respectively. To finish this fabrication, the Au/Si asymmetric dimer arrays are obtained via a lift-off process in acetone (see Figure 1). The geometrical parameters of Au/Si asymmetric dimer arrays are disk diameters of 130 nm (D) and 65 nm (d), a period of 300 nm (P), a gap of 10 nm (g), and a total height of 40 nm (h = 20 nm (Si) + 20 nm (Au)).

**Figure 2:**
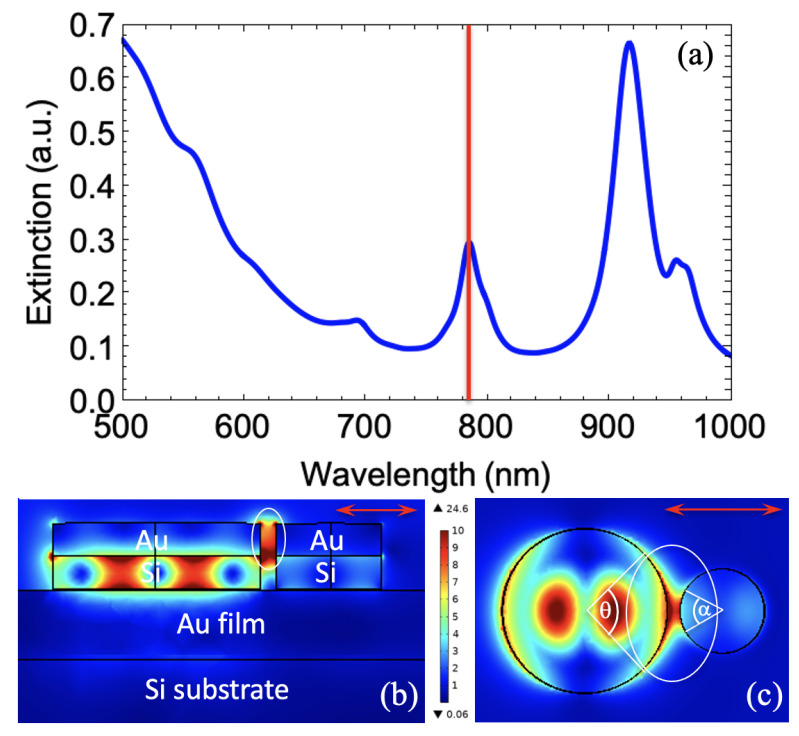
(**a**) Extinction spectrum calculated by FEM. The red line indicates the chosen wavelength of excitation. Electric field (E/E${}_{0}$) mappings calculated at the excitation wavelength of 785 nm: (**b**) cross-section view and (**c**) top view. In each mapping, the double red arrow corresponds to the direction of the electric field in the laser beam oriented along the dimer axis. The white ellipses indicate the strong electric fields zones accessible for thiophenol molecules (gold part) and taken into account for calculating the EF value (θ = 82${}^{{}^{\circ}}$ and α = 54${}^{{}^{\circ}}$ correspond to the part of the lateral surface to be taken into account for each nanodisk of dimer).

Furthermore, we have simulated the extinction spectrum of our SERS substrate in order to optimize the nanostructure geometry for obtaining a plasmonic resonance very close to the excitation wavelength of 785 nm. To do that, we used the COMSOL environment. The electromagnetic model contains the periodic system of asymmetric Au/Si disk dimers deposited on a continuous gold layer, and illuminated under normal incidence by a source polarized along the dimer axis (see Figure 1). The Maxwell equations are solved by the finite element method (FEM). The dielectric constants for gold and silicon are taken from the references [42,43]. Since the thickness of the gold film is larger than the skin-depth in metals (40 nm), extinction A is determined by the absorption (or extinction) A = 1 – R, where the reflection R is found by the COMSOL solution of the Maxwell equations. Figure 2a displays the extinction spectrum of hybrid asymmetric dimers calculated with the optimized dimensions for obtaining a plasmonic resonance very close to the excitation wavelength of 785 nm. From this extinction spectrum, we observe two plasmonic resonances at the positions of ∼785 nm and ∼915 nm, whose one resonance matches with the excitation wavelength. Moreover, the difference between these two plasmonic resonances is close to the Stokes shift of thiophenol. Finally, we calculate electric field mappings at this excitation wavelength of 785 nm displayed in Figure 2b,c.

### 2.2. Thiophenol Functionalization on Hybrid Asymmetric Dimers

In order to study the sensitivity of the hybrid asymmetric dimers, we employed thiophenol molecules for their ability of grafting (excellent adsorption) on surface of noble metals [44,45]. Firstly, we prepared solutions of thiophenol in ethanol with concentrations varying from 10${}^{-11}$ to 10${}^{-2}$ M. Then, for SERS experiments, the samples were dipped in the solution for 24 h followed by a thoroughly washing with pure ethanol for removing the unbound molecules. Finally, the samples were dried with nitrogen. For the classical Raman measurements in solution (reference), the thiophenol concentration of 1 M was used.

### 2.3. Raman Measurements

A Labram spectrophotometer (Horiba Scientific) with a spectral resolution of 1 cm^−1^ was employed. For all the SERS and Raman (reference) spectra, we have chosen an excitation wavelength of 785 nm ($\lambda_{exc}$), which is very close to a plasmon resonance of our hybrid asymmetric dimers (see Figure 2a). The acquisition time and the laser power have been set at 10 s and 3 mW, respectively. A half wave plate was employed to set the polarization of the incident laser beam along the dimer axis. For the SERS measurements, a microscope objective (×100, N.A. = 0.9) was used in order to focus the laser beam on the sample, and the Raman signal of the sample was detected by this same objective in a backscattering configuration. Furthermore, for Raman measurements in solution (reference), the same excitation wavelength and a macro-objective whose focal length is 40 mm (N.A. = 0.18) were employed. All recorded spectra have been divided by the acquisition time and the laser power for comparison purposes.

## 3. Results and Discussion

**Figure 3:**
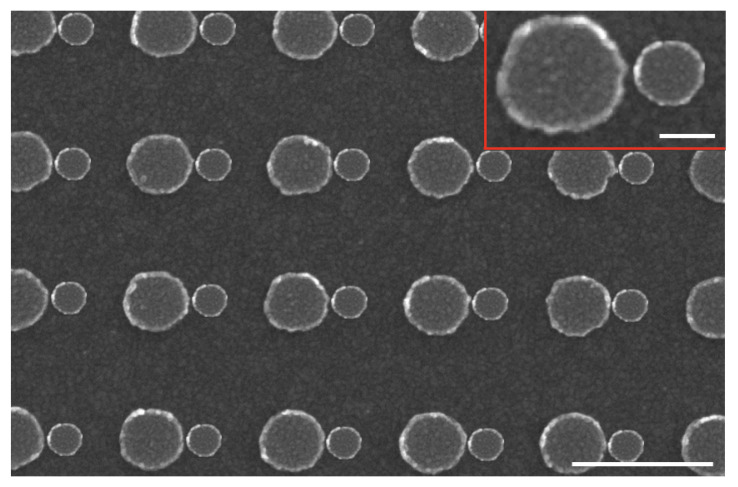
SEM picture of the hybrid asymmetric dimer array (scale bar = 300 nm). The inset corresponds to the zoom of an asymmetric dimer (scale bar = 50 nm).

Firstly, arrays of hybrid asymmetric dimers on gold film were realized according to the process detailed in Section 2.1. SEM images of these Au/Si asymmetric dimers are depicted in Figure 3. The dimensions of nanodisks for the dimer are *D* ∼ 130 nm, *d* ∼ 65 nm, *g* ∼ 10 nm and *h* ∼ 40 nm, and the period *P* = 300 nm.

**Figure 4:**
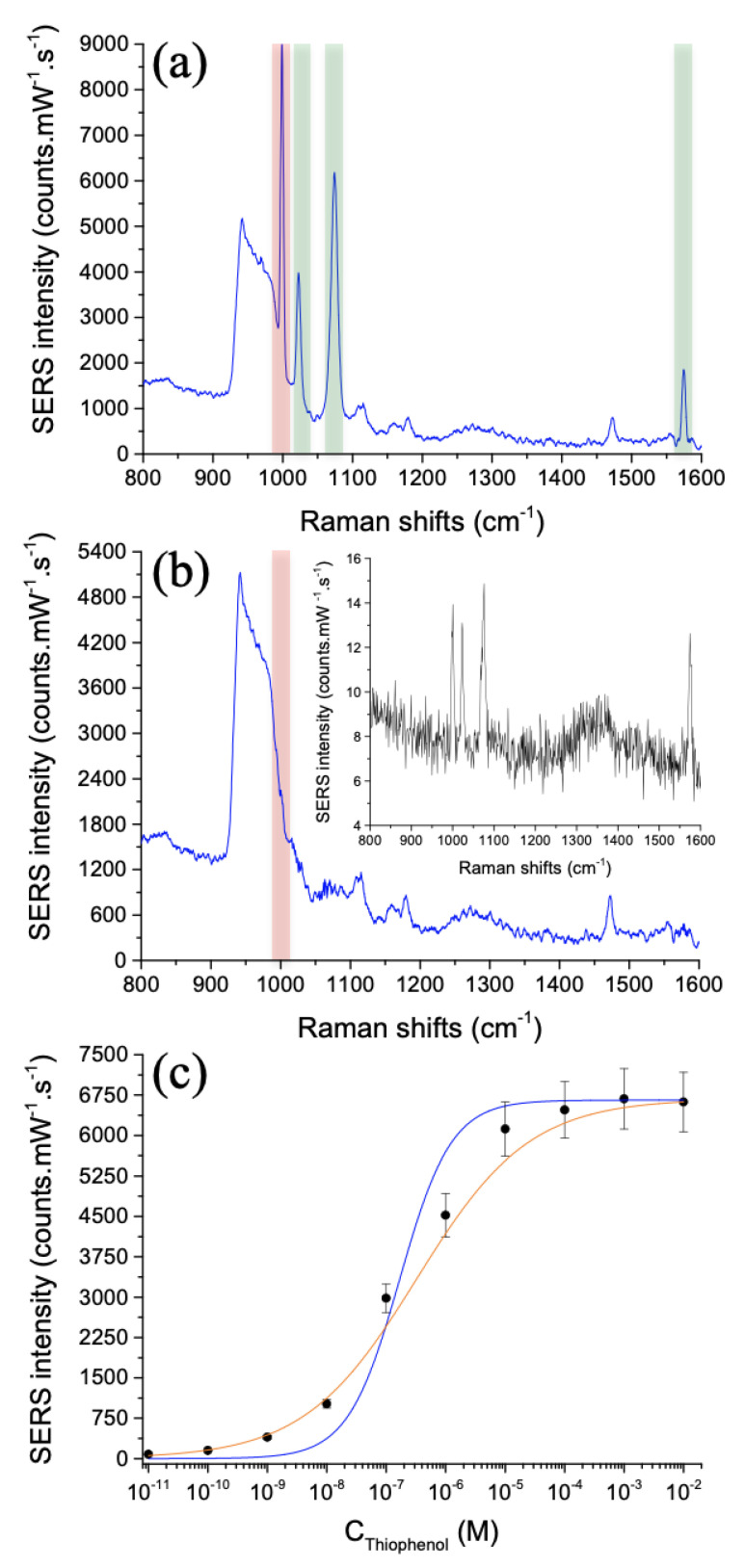
(**a**) SERS spectrum of thiophenol molecules (concentration of 1 mM) on hybrid asymmetric dimers. The Raman peak at 999 cm^−1^ is encapsulated in a red rectangle that we have chosen to use for assessing the sensitivity of hybrid asymmetric dimers, and the Raman peaks at 1023, 1075 and 1575 cm^−1^ are encapsulated in a green rectangle. (**b**) SERS spectrum of thiophenol molecules (concentration of 10 pM = limit of detection) on hybrid asymmetric dimers. The inset displays the SERS spectrum of thiophenol molecules (1 mM) recorded on the gold film (**c**) SERS intensity versus thiophenol concentration ($C_{S}$). The black points correspond to the experimental results, and the blue and orange curves correspond to the Langmuir and Hill models, respectively.

To assess the sensitivity of Au/Si asymmetric dimers, thiophenol molecules are grafted on these hybrid dimers by employing the functionalization process described in Section 2.2. Next, the SERS spectra have been recorded at the excitation wavelength of 785 nm. Figure 4 displays the SERS spectra of thiophenol molecules on Au/Si asymmetric dimers (see also the Figure S1 in Supplementary Information). From the SERS spectrum obtained for the concentration of 1 mM depicted in Figure 4a, four Raman peaks of thiophenol molecules [46,47,48] are well-observed like those at 999 cm^−1^ attributed to the C–H out-of-plane bending and ring out-of-plane deformation (called: γ(CH) and r-o-d), 1023 cm^−1^ corresponding to the ring in-plane deformation and C–C symmetric stretching (called: r-i-d and ν(CC)), 1075 cm^−1^ corresponding to the C–C symmetric stretching and C–S stretching (called: ν(CC) and ν(CS)), and at 1575 cm^−1^ corresponding to the C–C symmetric stretching mode (called: ν(CC)). Other Raman peaks are observed due to the presence of silicon in the nanostructures and correspond to the multi-phonon peaks of Si located at 900–980 cm^−1^ [49,50].

In order to determine this sensitivity of detection, the Raman peak at 999 cm^−1^ was chosen for its highest value of SERS intensity. SERS spectra have been recorded for each thiophenol concentration and we plotted the SERS intensity as a function of thiophenol concentration (see Figure 4c, and also the Figure S1 in Supplementary Information). From this Figure 4c, a limit of detection was found and equals to a thiophenol concentration of 10 pM. Then, we have fitted the experimental results with a Langmuir model expressed as follows [38,39,40,41]:

(1) $$\begin{array}{c} I=I_{max}\frac{KC_{S}}{1+KC_{S}} \end{array}$$

where $C_{S}$ corresponds to the studied concentration of thiophenol, *I* represents the SERS intensity at the concentration $C_{S}$. $I_{max}$ represents the maximum value of the SERS intensity corresponding the formation of the self-assembled monolayer of thiophenol. Finally, *K* is the adsorption constant. A good agreement is obtained between the experimental results and the fitting curve using the Langmuir model (see the blue curve in Figure 4c), where the value of *K* is extracted and equals to 6.0 × 10${}^{6}$ M${}^{-1}$. This value of *K* is higher than this obtained with Klarite SERS substrates (arrays of gold inverted pyramid structures) for thiophenol detection (*K* = 1.1 × 10${}^{6}$ M${}^{-1}$) [40], and indicates a good adsorption affinity of thiophenol molecules on the gold part of the hybrid asymmetric dimer array. Furthermore, we can calculate the enhancement factor (EF) of these hybrid asymmetric dimers by taking into account this value of *K*. Thus, the EF expression is as follows [38]:

(2) $$\begin{array}{c} EF=\frac{I_{SERS}}{I_{Raman}}\times \frac{C_{Raman}V\left(1+KC_{S}\right)}{KC_{S}n_{max}A} \end{array}$$

where $I_{SERS}$, $I_{Raman}$ represent the SERS and the normal Raman intensities, respectively. $C_{Raman}$ corresponds to the thiophenol concentration in solution (in ethanol; here $C_{Raman}$ = 1 M), *V* represents the scattering volume producing the normal Raman signal, and *A* is the area illuminated by the laser producing the SERS signal. $n_{max}$ is the surface coverage of a thiophenol monolayer, which is around 5.44 × 10${}^{-10}$ mol/cm^2^ [51]. The scattering volume is calculated by this expression: *V* = $A_{sca}$ × *H*, where $A_{sca}$ is the scattering area corresponding to the disk area whose diameter is 5.3 µm at the excitation wavelength of 785 nm, and *H* is the scattering height which is equal to 32 µm at this same wavelength of excitation [52,53]. Here, *V* is equal to 7.04 × 10${}^{-13}$ L. The area *A* is determined by the following expression: *A* = *N* × $S_{lat}$, where *N* corresponds to the number of dimers (here ∼16) illuminated in the laser spot whose the size is ∼1 µm^2^ at the excitation wavelength of 785 nm, and $S_{lat}$ corresponds to the sum of the lateral surface parts (gold part) of each nanodisk displayed in Figure 2b,c (see the white ellipses) corresponding to the zones of strong electric field accessible for thiophenol molecules (here, $S_{lat}$ = 4.1 × 10${}^{-10}$ cm^2^). Besides, no significant SERS signal is observed from the gold film (see the inset of Figure 4b). Thus, the EF value is 5.2 × 10${}^{10}$ at the detection limit of 10 pM (see Table 1 for all the parameters of the EF calculation).

Moreover, this EF value is higher than those obtained with gold dimer arrays on gold film (50 nm), where the dimer is composed of two disks (or rectangles or bowties) with a diameter of 140 nm, a gap of 20 nm and a height of 40 nm (EF = 3 × 10${}^{5}$–4 × 10${}^{5}$) [24], and with 3D donut-like gold nanorings on a gold film (EF = 3.84 × 10${}^{7}$) [54]. Furthermore, we have fitted the experimental results with another model called Hill equation, and defined it as follows [55,56]:

(3) $$\begin{array}{c} I=I_{max}\frac{1}{1+{\left(\frac{k}{C_{S}}\right)}^{n}} \end{array}$$

where $C_{S}$, *I*, and $I_{max}$ have the same correspondence than with the Langmuir model. *k* represents the concentration of molecules generating half coverage, and *n* the Hill coefficient. A nice agreement is achieved between the experimental results and the fitting curve using the Hill Equation (see the orange curve in Figure 4c), where the values of *k* and *n* are extracted and equal to 3.3 × 10${}^{-7}$ M and 0.46, respectively. This value of *k* = 3.3 × 10${}^{-7}$ M confirms the high affinity of thiophenol molecules towards the gold surface. However, the value *n* = 0.46 is inferior to 1 indicating that thiophenol molecules already bound to the gold surface reduces the affinity of the incoming molecules to this surface [55].

## 4. Conclusions

In this paper, we experimentally demonstrated the high sensitivity of this Au/Si asymmetric dimer array on gold film for SERS sensing of thiophenol molecules, which are well-known for their excellent adsorption on noble metals and serving here as a proof-of-concept. For this sensing, a detection limit of 10 pM was reached as well as an adsorption constant *K* of 6 × 10${}^{6}$ M${}^{-1}$. This value of *K* has indicated that the adsorption affinity of thiophenol molecules on gold surface was good. Moreover, this high adsorption affinity of thiophenol molecules on gold surface was confirmed by the value *k* of 3.3 × 10${}^{-7}$ M obtained with the Hill equation. Furthermore, the sensitivity was also compared to the experimental results in the literature obtained for gold dimers of different geometries and gold nanorings both on a gold film. The EF value was calculated by taking into account the adsorption constant *K*, and has achieved 5.2 × 10${}^{10}$ with our SERS substrates at the detection limit of 10 pM, which was higher than EFs of gold nanostructures cited above. In addition, this approach can be applied to several SERS substrates allowing a prescriptive estimate of their relative performance as sensor and to probe the affinity of substrates for a target analyte. Moreover, this prescriptive estimate leads to higher predictability of SERS activity of molecules, which is also a key point for the development of sensors for a broad spectrum of analytes. Thus, all of these considerations, such as the design of plasmonic nanostructures (i.e., zones of strong electric field, and the plasmonic resonances), and the adsorption affinity of molecules toward the metallic surface (here gold), are significant and to be taken into account for achieving the maximum SERS signal.

## Figures and Tables

**Table 1:** For the calculation of EF at the excitation wavelength of 785 nm with Equation (2), all the parameters are displayed. $I_{Raman}$ is obtained from our Raman spectrum displayed in the Figure S2 in Supplementary Information.

Parameters for EF	Values
Raman peak (cm^−1^)	999
$I_{SERS}$ (counts·mW${}^{-1}$·s${}^{-1}$)	78
$I_{Raman}$ (counts·mW${}^{-1}$·s${}^{-1}$)	79
$C_{Raman}$ (M)	1
$C_{S}$ (M)	10${}^{-11}$
*K* (M${}^{-1}$)	6.0 × 10${}^{6}$
*V* (*L*)	7.04 × 10${}^{-13}$
*A* (cm^2^)	4.1 × 10${}^{-10}$
$n_{max}$ (mol/cm^2^)	5.44 × 10${}^{-10}$

## Supplementary Materials

The following are available online at https://www.mdpi.com/article/10.3390/nano11061521/s1, Figure S1: SERS spectra of thiophenol recorded at the excitation wavelength of 785 nm for the concentrations of 10${}^{-2}$ M and from 10${}^{-4}$ M to 10${}^{-10}$ M, Figure S2: Raman spectra of a thiophenol solution of 1 M (thiophenol + ethanol) and the pure ethanol recorded at the excitation wavelength of 785 nm.
