# Supplementary material for: SERS Amplification in Au/Si Asymmetric Dimer Array Coupled to Efficient Adsorption of Thiophenol Molecules
Source: Nanomaterials (Basel). 2021 Jun 8;11(6):1521. doi: 10.3390/nano11061521 (PMC8227605; doi:10.3390/nano11061521)
Supplement: Supplementary file 1 [file nanomaterials-11-01521-s001.zip › nanomaterials-1244601-supplementary.pdf]

Supplementary Information

SERS amplification in Au/Si asymmetric dimer array coupled to efficient adsorption of thiophenol molecules

Grégory Barbillon[a], Andrey Ivanov[b], Andrey K. Sarychev[b]

[a]*EPF-Ecole d'Ingénieurs, 92330 Sceaux, France.*
[b]*Institute for Theoretical and Applied Electrodynamics, Russian Academy of Sciences, 125412, Moscow, Russia.*

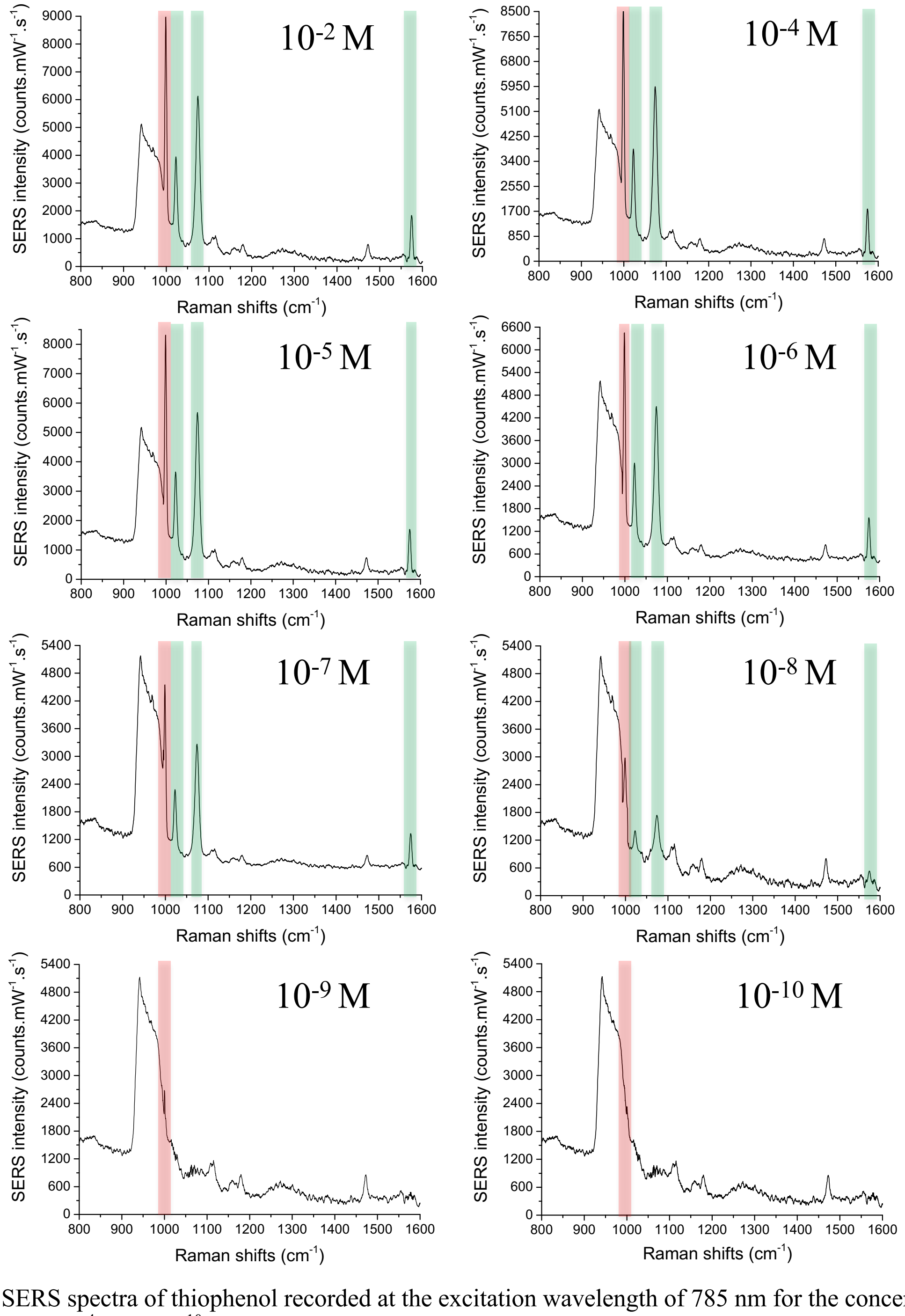

Figure S1. SERS spectra of thiophenol recorded at the excitation wavelength of 785 nm for the concentrations of 10^{-2} M and from 10^{-4} M to 10^{-10} M, where the four Raman peaks are displayed, especially the Raman peak at 999 cm^{-1} which is encapsulated in a red rectangle, and that we have chosen to use for assessing the sensitivity of hybrid asymmetric dimers.

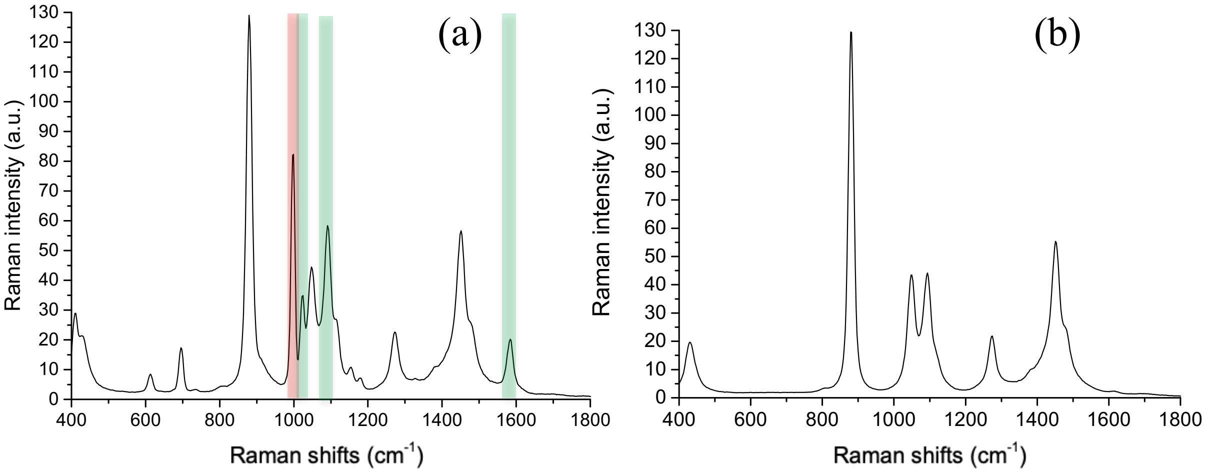

Figure S2. Raman spectra of (a) a thiophenol solution of 1 M (thiophenol + ethanol) and (b) the pure ethanol recorded at the excitation wavelength of 785 nm.
